# Children in Tokyo Have a Long Sustained Axial Length from Age 3 Years: The Tokyo Myopia Study

**DOI:** 10.3390/jcm11154413

**Published:** 2022-07-29

**Authors:** Tomoki Maruyama, Erisa Yotsukura, Hidemasa Torii, Kiwako Mori, Mikako Inokuchi, Mitsuaki Tokumura, Debabrata Hazra, Mamoru Ogawa, Akiko Hanyuda, Kazuo Tsubota, Toshihide Kurihara, Kazuno Negishi

**Affiliations:** 1 Department of Ophthalmology, Keio University School of Medicine, Shinjuku-ku, Tokyo 160-8582, Japan; tomoki.maruyama@keio.jp (T.M.); erisa.yotsuku@icloud.com (E.Y.); morikiwako@gmail.com (K.M.); saraswati@a3.keio.jp (D.H.); mamoogawa@gmail.com (M.O.); akikohanyuda@gmail.com (A.H.); tsubota@z3.keio.jp (K.T.); kazunonegishi@keio.jp (K.N.); 2 Laboratory of Photobiology, Keio University School of Medicine, Shinjuku-ku, Tokyo 160-8582, Japan; 3 Health Center, Keio University, Kohoku-ku, Yokohama-shi 223-8521, Japan; mikakoin@ybb.ne.jp (M.I.); tokumura@z2.keio.jp (M.T.); 4 Department of Pediatrics, Keio University School of Medicine, Shinjuku-ku, Tokyo 160-8582, Japan; 5 Tsubota Laboratory, Inc., Shinjuku-ku, Tokyo 160-0016, Japan

**Keywords:** myopia, ocular biometry, axial length

## Abstract

Background: myopia prevalence is high among Japanese schoolchildren, but the underlying causes are unclear. Objective: To examine the distributions of ocular biometry and refraction and their associations with lifestyle variables among Japanese schoolchildren. Methods: This cross-sectional school-based study included 2140 children aged 3–14 years in Tokyo, Japan, and evaluated the distributions under non-cycloplegic conditions and the associated environmental factors. Results: The prevalence of spherical equivalent (SE) ≤−0.75 diopter among preschoolers (aged 3–6 years), elementary school students (aged 6–11 years), and junior high school students (aged 12–14 years) was 49.7%, 72.4%, and 87.7%, respectively. Multiple linear regression analyses showed that the time spent using digital devices was associated positively with lens thickness (β = 0.010; *p* < 0.050) but not SE, axial length, or vitreous chamber depth. The time spent reading was associated negatively with lens thickness (β = −0.012; *p* < 0.050), SE (β = −0.152; *p* < 0.010), axial length (β = 0.110; *p* < 0.001), and vitreous chamber depth (β = 0.110; *p* < 0.001). Conclusions: The data indicated that almost half of preschoolers may be myopic. The association with the lens thickness differed depending on the type of near work performed.

## 1. Introduction

The prevalence of myopia is increasing worldwide, especially in East Asia, and has become a major public health problem [1,2]. We previously investigated the prevalence of myopia among elementary and junior high school students in Tokyo, Japan, and reported that the prevalence was 76.5% among elementary school students, and 94.9% among junior high school students [3]. However, it remains unclear why the prevalence of myopia is so high among Japanese children and how myopia develops [4].

Refraction is affected by the corneal power, lens power, and axial length (AL) [5]. Myopia often progresses in school-age children, and changes in the balance of each ocular component with growth may be related to the progression of myopia [6]. The sex- and age-specific mean values of the ocular components have been investigated among children in many countries and regions, including Taiwan [7], Hong Kong [8], mainland China [9], and Germany [10], using school-based cross-sectional designs. However, to the best of our knowledge, no studies have reported sex- and age-specific summary statistics of these various measurements in a general population with a wide age range using a school-based design in Japanese children.

Previous epidemiologic studies have suggested that several lifestyle factors may be associated with myopia, including time spent outdoors [11], use of digital devices [12], reading [13], and watching television [14,15,16]. Regarding near work, the underlying mechanisms linking risk factors to myopia progression are poorly understood. Furthermore, when investigating the association between near work and myopia, it is better to focus the crystalline lens under non-cycloplegic natural conditions.

Although some theories about the etiology of myopia have been forwarded including accommodative-lag theory [17,18], peripheral hyperopic defocus theory [19], and light environment theory [20], the origin is unknown. Several previous studies have examined the associations between lifestyle factors and ocular components to obtain clues to better understand the pathogenesis of myopia [21,22,23], but the previous studies had limitations, i.e., only adults were included [21] or few near work-related variables were examined [21,22].

In the current study, we report the detailed distributions of the ocular biometry and refraction among Japanese children with a wide age range using a school-based design and evaluated the associations between refraction and environmental factors including near work and ocular biometry under non-cycloplegic natural conditions.

## 2. Materials and Methods

This cross-sectional study is a part of the Tokyo Myopia Study, a school-based observational and longitudinal study [3]. The present study was based on data from a baseline survey.

**Figure 1:**
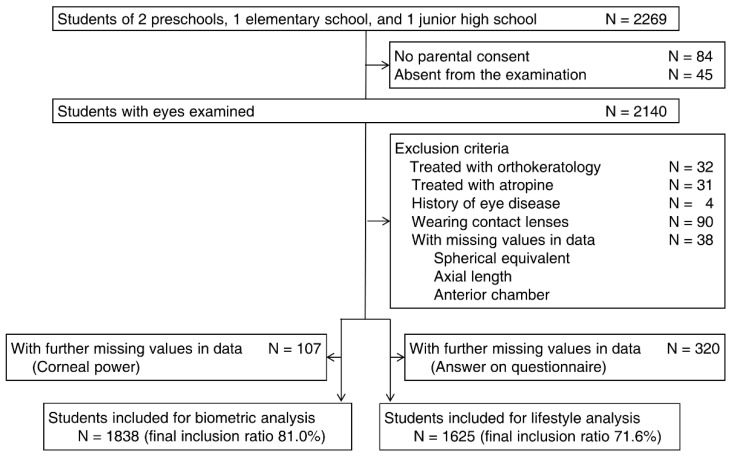
Study flowchart of participant inclusion and exclusion.

Detailed inclusion and exclusion procedures are described in the flowchart (Figure 1). Briefly, all students from two preschools, one elementary school, and one junior high school were invited to participate. The survey took place in 2019 in the preschools and elementary school and in 2017 in the junior high school. These schools were selected because they are conveniently located in Tokyo so students were convenient samples. No children were invited repeatedly to participate in the current study. Students without serious eye diseases (adenovirus infection, *n* = 1; conjunctivitis, *n* = 1; trichiasis, *n* = 1; morning glory syndrome, *n* = 1; total *n* = 4) or missing values were included in the analyses. We excluded children treated with orthokeratology or atropine and who wore contact lenses during the measurements. Hence, data from 1838 children were used to analyze the distribution of ocular biometry, and data from 1625 children were used to assess the relationships between lifestyle variables and specific ocular biometry. The final inclusion percentages of the two parameters were 81.0% and 71.6%, respectively. All children underwent bilateral eye examinations and the results from the right eyes are presented because of the high correlation with the fellow eyes (correlation coefficients of both eyes: refraction, 0.827, *p* < 0.001; axial length, 0.975, *p* < 0.001).

The baseline survey was conducted during annual health check-ups. The details of the current study have been reported previously [3]. The height and weight were measured, and the body mass index (BMI) was calculated. Non-cycloplegic refraction was measured using HOYA iTrace Surgical Workstation (Tracey Technologies, Houston, TX, USA). The AL, corneal thickness, anterior chamber depth, lens thickness, and corneal radius curvature (CR) were measured using the IOLMaster 700 (Carl Zeiss Meditec AG, Jena, Germany). The AL was measured 10 times, and the averaged value was used. The spherical equivalent, vitreous chamber depth, and corneal power, AL–CR curvature (AL/CR) ratio were calculated. Myopia was defined as a spherical equivalent of less than or equal to −0.5 diopters (D) in accordance with the international classification of myopia proposed by the International Myopia Institute in 2019 [24]. The students and their parents completed the questionnaire that we used previously [3] to record the time spent outdoors, on near work, sleeping, using digital devices including computers/smartphones/tablets/handheld game consoles, studying, and parental history of myopia. We set the maximal value for time spent outdoors as 180 min daily, because the association between outdoor activities and suppression of myopia onset and progression may reach a plateau at that point [11].

All statistical analyses were performed using R version 4.0.3 (R Foundation for Statistical Computing, Vienna, Austria). Two-tailed *P* values less than 0.05 were considered statistically significant. The association between each of the outcome variables (refraction and ocular biometry) and each of the possible confounders (age, sex, BMI, time spent outdoors, television viewing, use of digital devices, reading or studying, and the number of myopic parents) was examined using simple linear regression analysis. The associations also were examined using multiple linear regression analysis, because simple linear regression analysis showed that all of these variables were associated significantly (*p* < 0.05). In multiple regression analysis, all variables were included in the forced entry manner to examine associations between lifestyle variables and outcomes with adjustment for other variables.

## 3. Results

**Figure 2:**
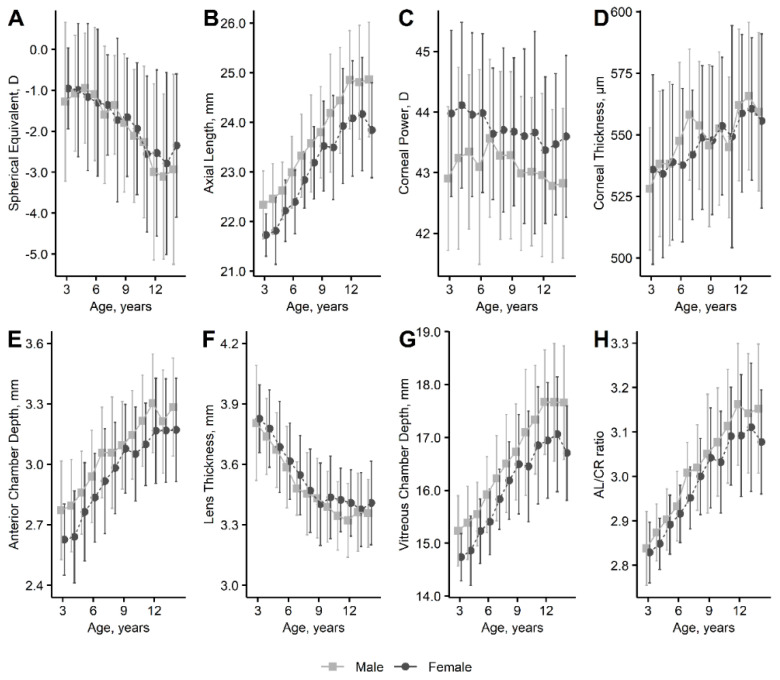
Sex- and age-specific distribution of ocular biometry. The means and 95% confidence intervals are shown. (**A**), Spherical equivalent. (**B**), Axial length, (**C**), Corneal power. (**D**), Corneal thickness. (**E**), Anterior chamber depth. (**F**), Lens thickness. (**G**), Vitreous Chamber depth. (**H**), AL/CR. D = diopter; AL/CR ratio = axial length–corneal radius ratio.

Table 1 shows the baseline characteristics of the participants (data based on gender are available in Supplementary Table S1a–d). The prevalence of myopia was 60.2% among preschoolers, 82.2% among elementary school students, and 92.8% among junior high school students. The percentages of patients with −0.75 D or less of myopia were 49.7%, 72.4%, and 87.7%, respectively. The sex- and age-specific mean values and standard deviations are shown in Figure 2, and the numeric values are available in Supplementary Tables S2–S9. The spherical equivalent decreased with age from ages 3 to 12 years (mean ± standard deviations for ages 3 and 12 years, −1.16 ± 1.64 D and −2.82 ± 2.12 D, respectively). The Als increased from ages 3 to 12 years (22.12 ± 0.67 mm and 24.57 ± 1.12 mm, respectively). The lens became thinner from ages 3 to 12 years (3.81 ± 0.25 mm and 3.35 ± 0.18 mm, respectively). The vitreous chamber depth increased from ages 3 to 12 years (15.05 ± 0.63 mm and 17.41 ± 1.08 mm, respectively). The AL/CR ratio increased from ages 3 to 12 years (2.84 ± 0.08 and 3.14 ± 0.14, respectively).

Table 2 shows the lifestyle characteristics of each school group (data based on gender are available in Supplementary Table S10a–d). The mean time spent daily using digital devices/reading among preschoolers, elementary school students, and junior high school students were, respectively, 25.3/30.8, 40.5/68.4, and 115.4/74.7 min/day.

Table 3 shows the results of a simple linear regression analysis performed to estimate the association between ocular components and lifestyle variables.

Table 4 shows the results of a multiple linear regression analysis performed to estimate the association between ocular components and lifestyle variables with adjustment for all possible confounders (i.e., age, sex, BMI, time spent outdoors, television viewing, use of digital devices, reading or studying, and the number of myopic parents). The results indicated that a more myopic spherical equivalent was associated significantly with older age (coefficient β = −0.208; −0.244 to −0.172 D), shorter time spent outdoors (β = 0.131; 0.021 to 0.241 D), longer time spent reading (β = −0.152; −0.254 to −0.049 D), and higher number of myopic parents (β = −0.305; −0.425 to −0.185 D). Moreover, based on the results, longer AL was associated with older age (β = 0.254; 0.236 to 0.271 mm), male sex (β = 0.622; 0.531 to 0.712 mm), shorter time spent outdoors (β = −0.058; −0.113 to −0.003 mm), longer time spent reading (β = 0.110; 0.059 to 0.161 mm), and higher number of myopic parents (β = 0.233; 0.174 to 0.293 mm). The results also showed that thicker lenses were associated with younger age (β = −0.042; −0.046 to −0.038 mm), female sex (β = −0.032; −0.052 to −0.013 mm), higher BMI (β = 0.007; 0.002 to 0.012 mm), longer time spent using digital devices (β = 0.010; 0.001 to 0.020 mm); shorter time spent reading (β = −0.012; −0.023 to −0.001 mm), and lower number of myopic parents (β = −0.016; −0.029 to −0.003 mm). The results also showed that longer vitreous chamber depth was associated with older age (β = 0.245; 0.228 to 0.262 mm), male sex (β = 0.550; 0.462 to 0.638 mm), lower BMI (β = −0.028; −0.050 to −0.006 mm), shorter time spent outdoors (β = −0.058; −0.111 to −0.005 mm), longer time spent reading (β = 0.110; 0.061 to 0.160 mm), and higher number of myopic parents (β = 0.212; 0.154 to 0.270 mm).

## 4. Discussion

The current study showed the various sex- and age-specific distributions of ocular component parameters and refraction in detail and analyzed the associations between these components and lifestyle variables in nearly 2000 Japanese children. It is noteworthy that the AL elongation seems to slow around the age of 12 years. The current results also indicated that the associations between near work and lens thickness may vary with the type of near work.

**Figure 3:**
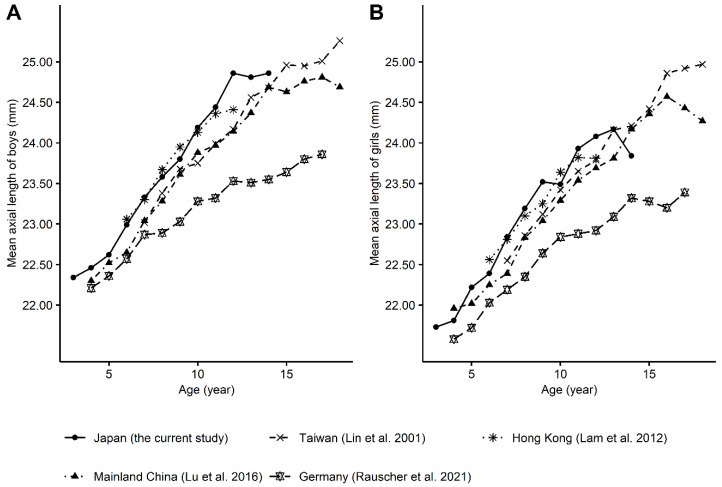
Sex- and age-specific distribution of the mean axial length (AL) from the current study and other school-based cross-sectional studies [7,8,9,10]. The mean ALs as a function of age are plotted for, (**A**), boys and, (**B**), girls using data from the current study and other school-based cross-sectional studies. For AL measurements, A-scan ultrasonography was used in the Taiwanese study, and optical biometry was used in the other studies.

Of particular interest in the current study was the sex- and age-specific distribution of AL, which seems to disagree with those in previous cross-sectional school-based studies [7,9,10,25]. However, the mean values of corneal thickness, corneal refractive power, anterior chamber depth, and lens thickness agreed with previous studies [7,10,25]. It has been suggested that AL elongation may slow by the age of 10 to 13 years, with the mean AL stabilizing around 23 to 24 mm in non-myopic eyes among boys and girls, whereas, in myopic eyes, the AL elongates more than in non-myopic eyes, and the elongation starts to level off around the age of 14 to 16 years, with the mean AL stabilizing around 25.5 mm among boys and 24.0 mm among girls [26,27,28]. Although there are no data from children older than 14 years, the current results differ from these typical patterns in that the AL elongations seem to slow by the age of 13 years among boys and girls in a non-myopic population, but the mean ALs stabilize around 25.0 mm among boys and 24.0 mm among girls as in a myopic population (Figure 3). Figure 3 also includes data extracted from other school-based cross-sectional studies, which provided tables showing sex- and age-specific mean AL as numeric values with two decimal places, i.e., studies from Taiwan [7] (total number of AL measurement, 10,570; age range, 7 to 18 years); Hong Kong [8] (*n* = 2651; ages 6 to 12 years); mainland China [9] (*n* = 5972; ages 4 to 18 years); and Germany [10] (*n* = 1744; ages 4 to 17 years). Although age differences in each cross-sectional study are susceptible to cohort biases and inter-study heterogeneity may reflect differences in research settings, Figure 3 may also show that AL elongation patterns differ across regions and countries, possibly with a rapid change in Japan and Hong Kong [8] compared with Taiwan [7], mainland China [9], and Germany [10]. Given that Chinese ethnicity accounts for 92% of the Hong Kong population [29], possible differences in elongation patterns between Hong Kong [8] and mainland China [9] may be due to the environments, including rural or urban, rather than genetic ancestry. Confirming these findings could be important, as modifiable factors may affect AL elongation patterns in school-age populations.

The current study is noteworthy in that we focused on the lens thickness and measured it without using cycloplegia. Saw et al. [22] and Liu et al. [23] investigated the associations between lens thickness and lifestyle factors, such as the number of books read/week [22] or time using electronic devices [23] among children, and no significant associations were detected. To the best of our knowledge, the current study is the largest childhood population-based study to evaluate the associations between lens thickness and lifestyle factors. Our results suggested that the time spent using digital devices is associated positively with the lens thickness and that the time spent reading is associated negatively, after adjusting for confounders. This difference in associations may explain why some specific types of near work, such as reading, are associated with myopia [13], although other near work, such as using digital devices, are not [30,31], in light of one of the theories of myopia pathogenesis. The accommodative-lag theory assumes that accommodative lag during near work causes hyperopic blur in the macula and may elongate the AL [17,18]. Based on this theory, a decrease in the lens thickness during reading may cause hyperopic blur in the macula; an increase in the lens thickness during the use of digital devices may compensate for a near work-induced accommodative lag. Collectively, the current study proposes that lens thickness may be involved in the associations between myopia and the type of near work. We would like to evaluate whether the lens thickness changes with the type of near work and to objectively measure lifestyle factors including the time spent outdoors in a future study, because the underlying reasons for the findings remain uncertain, i.e., that the time spent using digital devices might be associated positively with the lens thickness and that the time spent reading might be associated negatively.

The current study had several limitations. First, the refractive data were collected without cycloplegia because one of the main purposes of the current study was to examine the distributions of lens thicknesses in the natural state and the association with lifestyle variables. Second, the study participants were children from preschools and schools in Tokyo. Third, the lifestyle information was obtained by self-reported questionnaires; thus, the lifestyle data may have been subjected to recall bias. Fourth, the current study was a cross-sectional study, so the causality is unknown.

## 5. Conclusions

In conclusion, to our knowledge, this is the first large-scale study to describe sex- and age-specific various comprehensive ocular component data and refraction with a wide age range using a school-based design and analyzing their associations with environmental factors in Japanese children. Of note, we found that the prevalence of myopia among preschoolers was considered to be almost half. The effect of the lens thickness differed depending on the type of near work and may become a clue to investigating the relationship between near work and myopia.

## Figures and Tables

**Table 1:** Ocular characteristics based on school groups.

	Preschool (Ages 3 to 6 Years)	Elementary School (Ages 6 to 11 Years)	Junior High School (Ages 12 to 14 Years)
Number	596	663	579
Male	53.0%	49.3%	65.6%
Spherical equivalent (diopters)	−1.02 ± 1.53	−1.77 ± 1.72	−2.86 ± 2.12
≥0.5 diopter (hyperopia)	7.1%	2.6%	1.2%
−0.5 to 0.5 diopter (emmetropia)	32.7%	15.2%	6.0%
≤−0.5 diopter (myopia)	60.2%	82.2%	92.8%
≤−0.75 diopter	49.7%	72.4%	87.7%
Axial length (mm)	22.39 ± 0.73	23.46 ± 1.07	24.57 ± 1.16
Corneal power (diopter)	43.57 ± 1.47	43.48 ± 1.37	43.07 ± 1.29
Corneal thickness (µm)	538 ± 32	549 ± 31	561 ± 31
Anterior chamber depth (mm)	2.80 ± 0.25	3.04 ± 0.25	3.23 ± 0.26
Lens thickness (mm)	3.69 ± 0.21	3.47 ± 0.20	3.36 ± 0.18
Vitreous chamber depth (mm)	15.36 ± 0.70	16.41 ± 1.05	17.41 ± 1.11
Axial length–corneal radius ratio	2.89 ± 0.07	3.02 ± 0.12	3.13 ± 0.14

The data are expressed as percentages or means ± standard deviations.

**Table 2:** Lifestyle characteristics based on school groups.

	Preschool (Ages 3 to 6 Years)	Elementary School (Ages 6 to 11 Years)	Junior High School (Ages 12 to 14 Years)
Number	526	543	556
Age, years	4.8 ± 0.9	8.4 ± 1.7	12.9 ± 0.8
Male	54.4%	49.5%	65.6%
Body mass index, kg/m^2^	15.8 ± 1.5	16.4 ± 2.1	18.7 ± 2.4
Time spent, min/day			
Outdoors	73.7 ± 43.7	71.5 ± 45.7	72.2 ± 56.1
Watching television	92.3 ± 61.8	85.2 ± 58.1	80.7 ± 60.9
Use of digital devices	25.3 ± 37.9	40.5 ± 47.9	115.4 ± 88.5
Reading	30.8 ± 23.3	68.4 ± 65.6	74.7 ± 60.0
Number of myopic parents			
0 (none)	24.1%	15.5%	13.0%
1 (either)	35.2%	33.7%	45.1%
2 (both)	40.7%	50.8%	41.9%

The data are expressed as means ± standard deviations or as percentages.

**Table 3:** The associations between ocular parameters and lifestyle by simple linear regression analysis.

	Spherical Equivalent, D	Axial Length, mm	Lens Thickness, mm	Vitreous Chamber Depth, mm
Age (years)	−0.224 *** (−0.249 to −0.198)	0.268 *** (0.255 to 0.281)	−0.039 *** (−0.042 to −0.036)	0.254 *** (0.241 to 0.267)
Sex; male, 1; female, 0	−0.297 ** (−0.492 to −0.101)	0.791 *** (0.664 to 0.918)	−0.058 *** (−0.082 to −0.035)	0.705 *** (0.583 to 0.827)
Body mass index (kg/m^2^)	−0.158 *** (−0.197 to −0.118)	0.209 *** (0.184 to 0.235)	−0.026 *** (−0.031 to −0.022)	0.190 *** (0.165 to 0.214)
Time spent (h/day)				
Outdoors	0.164 ** (0.045 to 0.283)	−0.052 (−0.132 to 0.029)	−0.005 (−0.019 to 0.009)	−0.055 (−0.132 to 0.023)
Watching television	0.104 * (0.008 to 0.201)	−0.075 * (−0.141 to −0.010)	0.012 * (0.001 to 0.024)	−0.073 * (−0.136 to −0.011)
Use of digital devices	−0.351 *** (−0.428 to −0.274)	0.410 *** (0.360 to 0.459)	−0.052 *** (−0.061 to −0.042)	0.382 *** (0.334 to 0.429)
Reading	−0.436 *** (−0.537 to −0.335)	0.447 *** (0.381 to 0.513)	−0.064 *** (−0.076 to −0.052)	0.430 *** (0.367 to 0.493)
Number of myopic parents	−0.377 *** (−0.507 to −0.247)	0.317 *** (0.230 to 0.405)	−0.028 *** (−0.043 to −0.012)	0.293 *** (0.209 to 0.377)

Thirty-two simple linear regression models were conducted. The spherical equivalent, axial length, lens thickness, or vitreous chamber depth were used as the outcome variables. *** *p* < 0.001, ** *p* < 0.010, and * *p* < 0.050.

**Table 4:** The associations between ocular parameters and lifestyle by multiple linear regression analysis.

	Spherical Equivalent, D	Axial Length, mm	Lens Thickness, mm	Vitreous Chamber Depth, mm
Age (years)	−0.208 *** (−0.244 to −0.172)	0.254 *** (0.236 to 0.271)	−0.042 *** (−0.046 to −0.038)	0.245 *** (0.228 to 0.262)
Sex; male, 1; female, 0	−0.172 (−0.354 to 0.010)	0.622 *** (0.531 to 0.712)	−0.032 ** (−0.052 to −0.013)	0.550 *** (0.462 to 0.638)
Body mass index (kg/m^2^)	0.036 (−0.009 to 0.080)	−0.018 (−0.040 to 0.004)	0.007 ** (0.002 to 0.012)	−0.028 * (−0.050 to −0.006)
Time spent (h/day)				
Outdoors	0.131 * (0.021 to 0.241)	−0.058 * (−0.113 to −0.003)	−0.007 (−0.019 to 0.004)	−0.058 * (−0.111 to −0.005)
Watching television	0.034 (−0.055 to 0.122)	−0.007 (−0.051 to 0.037)	0.002 (−0.008 to 0.011)	−0.006 (−0.049 to 0.037)
Use of digital devices	−0.037 (−0.126 to 0.051)	−0.010 (−0.054 to 0.034)	0.010 * (0.001 to 0.020)	−0.013 (−0.056 to 0.030)
Reading	−0.152 ** (−0.254 to −0.049)	0.110 *** (0.059 to 0.161)	−0.012 * (−0.023 to −0.001)	0.110 *** (0.061 to 0.160)
Number of myopic parents	−0.305 *** (−0.425 to −0.185)	0.233 *** (0.174 to 0.293)	−0.016 * (−0.029 to −0.003)	0.212 *** (0.154 to 0.270)
R-squared	0.179	0.559	0.345	0.541

Four multiple linear regression models were conducted. The spherical equivalent, axial length, lens thickness, or vitreous chamber depth were used as the outcome variables. The regression coefficients shown are unstandardized values. *** *p* < 0.001, ** *p* < 0.010, and * *p* < 0.050.

## Data Availability

The data analyzed during the current study are not publicly available due to institutional privacy guidelines but are available from the corresponding author on reasonable request.

## Supplementary Materials

The following supporting information can be downloaded at: https://www.mdpi.com/article/10.3390/jcm11154413/s1, Table S1a: Ocular characteristics based on sex (preschool); Table S1b: Ocular characteristics based on sex (elementary school); Table S1c: Ocular characteristics based on sex (junior high school); Table S1d: Ocular characteristics based on sex (overall); Table S2: Distribution of spherical equivalents in diopter, based on age and sex; Table S3: Distribution of axial lengths in mm, based on age and sex; Table S4: Distribution of corneal powers in diopter, based on age and sex; Table S5: Distribution of corneal thicknesses in µm, based on age and sex; Table S6: Distribution of anterior chamber depths in mm, based on age and sex; Table S7: Distribution of lens thicknesses in mm, based on age and sex; Table S8: Distribution of vitreous chamber depths in mm, based on age and sex; Table S9: Distribution of axial length–corneal radius ratios, based on age and sex; Table S10a: Lifestyle characteristics based on sex (preschool); Table S10b: Lifestyle characteristics based on sex (elementary school); Table S10c: Lifestyle characteristics based on sex (junior high school); Table S10d: Lifestyle characteristics based on sex (overall).
