# Supplementary material for: Children in Tokyo Have a Long Sustained Axial Length from Age 3 Years: The Tokyo Myopia Study
Source: J Clin Med. 2022 Jul 29;11(15):4413. doi: 10.3390/jcm11154413 (PMC9369597; doi:10.3390/jcm11154413)
Supplement: Supplementary file 1 [file jcm-11-04413-s001.zip › jcm-1794815-supplementary.pdf]

Table S1a. Ocular characteristics based on sex (preschool)

	Overall	Male	Female
Number	596	316	280
Age (years)	4.82 ± 0.88	4.86 ± 0.91	4.78 ± 0.84
Spherical equivalent (diopters)	−1.02 ± 1.53	−0.99 ± 1.40	−1.06 ± 1.66
≥ 0.5 diopter (hyperopia)	7.1%	4.1%	10.3%
−0.5 to 0.5 diopter (emmetropia)	32.7%	34.8%	30.4%
≤ −0.5 diopter (myopia)	60.2%	61.1%	59.3%
≤ −0.75 diopter	49.7%	50.0%	49.3%
Axial length (mm)	22.39 ± 0.73	22.63 ± 0.68	22.11 ± 0.70
Corneal power (diopter)	43.57 ± 1.47	43.20 ± 1.47	43.99 ± 1.34
Corneal thickness (μm)	538 ± 32	539 ± 32	537 ± 32
Anterior chamber depth (mm)	2.80 ± 0.25	2.85 ± 0.23	2.74 ± 0.25
Lens thickness (mm)	3.69 ± 0.21	3.69 ± 0.20	3.70 ± 0.22
Vitreous chamber depth (mm)	15.36 ± 0.70	15.56 ± 0.66	15.13 ± 0.67
Axial length–corneal radius ratio	2.89 ± 0.07	2.89 ± 0.07	2.88 ± 0.07

The data are expressed as percentages or means ± standard deviations.

Table S1b. Ocular characteristics based on sex (elementary school)

	Overall	Male	Female
Number	663	327	336
Age (years)	8.46 ± 1.73	8.51 ± 1.74	8.42 ± 1.72
Spherical equivalent (diopters)	−1.77 ± 1.72	−1.76 ± 1.71	−1.79 ± 1.73
≥ 0.5 diopter (hyperopia)	2.6%	1.2%	3.9%
−0.5 to 0.5 diopter (emmetropia)	15.2%	15.0%	15.5%
≤ −0.5 diopter (myopia)	82.2%	83.8%	80.6%
≤ −0.75 diopter	72.4%	73.1%	71.7%
Axial length (mm)	23.46 ± 1.07	23.75 ± 1.07	23.18 ± 1.00
Corneal power (diopter)	43.48 ± 1.37	43.22 ± 1.35	43.74 ± 1.35
Corneal thickness (µm)	549 ± 31	551 ± 29	546 ± 33
Anterior chamber depth (mm)	3.04 ± 0.25	3.09 ± 0.24	2.98 ± 0.25
Lens thickness (mm)	3.47 ± 0.20	3.44 ± 0.19	3.49 ± 0.21
Vitreous chamber depth (mm)	16.41 ± 1.05	16.67 ± 1.05	16.16 ± 0.98
Axial length–corneal radius ratio	3.02 ± 0.12	3.04 ± 0.12	3.00 ± 0.11

The data are expressed as percentages or means ± standard deviations.

Table S1c. Ocular characteristics based on sex (junior high school)

	Overall	Male	Female
Number	579	380	199
Age (years)	12.90 ± 0.81	12.93 ± 0.82	12.84 ± 0.79
Spherical equivalent (diopters)	−2.86 ± 2.12	−3.01 ± 2.16	−2.58 ± 2.04
≥ 0.5 diopter (hyperopia)	1.2%	0.5%	2.5%
−0.5 to 0.5 diopter (emmetropia)	6.0%	6.3%	5.5%
≤ −0.5 diopter (myopia)	92.8%	93.2%	92.0%
≤ −0.75 diopter	87.7%	89.7%	83.9%
Axial length (mm)	24.57 ± 1.16	24.84 ± 1.09	24.05 ± 1.11
Corneal power (diopter)	43.07 ± 1.29	42.86 ± 1.28	43.46 ± 1.22
Corneal thickness (μm)	561 ± 31	562 ± 31	559 ± 32
Anterior chamber depth (mm)	3.23 ± 0.26	3.27 ± 0.25	3.17 ± 0.26
Lens thickness (mm)	3.36 ± 0.18	3.35 ± 0.18	3.40 ± 0.18
Vitreous chamber depth (mm)	17.41 ± 1.11	17.67 ± 1.05	16.93 ± 1.05
Axial length–corneal radius ratio	3.13 ± 0.14	3.15 ± 0.14	3.10 ± 0.13

The data are expressed as percentages or means ± standard deviations.

Table S1d. Ocular characteristics based on sex (overall)

	Overall	Male	Female
Number	1838	1023	815
Age (years)	8.68 ± 3.46	9.02 ± 3.55	8.25 ± 3.30
Spherical equivalent (diopters)	−1.87 ± 1.95	−1.98 ± 2.00	−1.73 ± 1.87
≥ 0.5 diopter (hyperopia)	3.6%	1.9%	5.8%
−0.5 to 0.5 diopter (emmetropia)	18.0%	17.9%	18.1%
≤ −0.5 diopter (myopia)	78.4%	80.2%	76.1%
≤ −0.75 diopter	69.9%	72.1%	67.0%
Axial length (mm)	23.46 ± 1.33	23.81 ± 1.33	23.03 ± 1.20
Corneal power (diopter)	43.38 ± 1.40	43.08 ± 1.37	43.76 ± 1.33
Corneal thickness (μm)	549 ± 33	552 ± 32	546 ± 33
Anterior chamber depth (mm)	3.02 ± 0.31	3.08 ± 0.30	2.94 ± 0.30
Lens thickness (mm)	3.51 ± 0.24	3.48 ± 0.24	3.54 ± 0.24
Vitreous chamber depth (mm)	16.39 ± 1.27	16.70 ± 1.28	16.00 ± 1.14
Axial length–corneal radius ratio	3.01 ± 0.15	3.04 ± 0.16	2.98 ± 0.13

The data are expressed as percentages or means ± standard deviations.

Table S2. Distribution of spherical equivalents in diopters, based on age and sex

Age(years)	Overall		Male		Female	
	Mean	Standard deviation	Mean	Standard deviation	Mean	Standard deviation
3	−1.16	1.64	−1.28	1.94	−0.96	0.99
4	−1.03	1.52	−1.07	1.41	−0.99	1.63
5	−1.05	1.58	−0.95	1.34	−1.16	1.79
6	−1.18	1.71	−1.09	1.63	−1.30	1.80
7	−1.46	1.45	−1.60	1.68	−1.36	1.23
8	−1.53	1.64	−1.35	1.20	−1.72	2.00
9	−1.72	1.56	−1.79	1.69	−1.66	1.45
10	−2.02	1.61	−2.11	1.62	−1.94	1.61
11	−2.40	1.87	−2.26	1.85	−2.55	1.90
12	−2.82	2.12	−2.99	2.15	−2.53	2.03
13	−2.99	2.09	−3.10	2.02	−2.79	2.22
14	−2.76	2.18	−2.93	2.32	−2.35	1.75

Table S3. Distribution of axial lengths in mm, based on age and sex

Age(years)	Overall		Male		Female	
	Mean	Standard deviation	Mean	Standard deviation	Mean	Standard deviation
3	22.12	0.67	22.34	0.68	21.73	0.43
4	22.13	0.76	22.46	0.71	21.81	0.67
5	22.42	0.63	22.62	0.58	22.22	0.62
6	22.73	0.75	22.99	0.72	22.39	0.64
7	23.06	0.74	23.33	0.83	22.84	0.57
8	23.39	0.89	23.58	0.98	23.19	0.73
9	23.66	0.92	23.80	0.92	23.52	0.90
10	23.82	1.17	24.19	1.19	23.49	1.05
11	24.20	1.14	24.44	1.07	23.93	1.16
12	24.57	1.12	24.86	0.99	24.08	1.16
13	24.58	1.18	24.81	1.14	24.17	1.14
14	24.56	1.19	24.86	1.15	23.84	0.96

Table S4. Distribution of corneal powers in diopter, based on age and sex

Age (years)	Overall		Male		Female	
	Mean	Standard deviation	Mean	Standard deviation	Mean	Standard deviation
3	43.30	1.34	42.91	1.18	43.98	1.37
4	43.69	1.50	43.24	1.50	44.11	1.37
5	43.65	1.35	43.34	1.27	43.96	1.35
6	43.49	1.54	43.10	1.61	43.98	1.31
7	43.61	1.18	43.57	1.30	43.64	1.08
8	43.49	1.38	43.28	1.39	43.71	1.35
9	43.49	1.31	43.29	1.38	43.68	1.22
10	43.31	1.39	42.99	1.27	43.60	1.44
11	43.32	1.48	43.02	1.22	43.66	1.67
12	43.11	1.31	42.96	1.34	43.37	1.21
13	43.03	1.27	42.78	1.26	43.47	1.16
14	43.06	1.31	42.83	1.24	43.60	1.33

Table S5. Distribution of corneal thicknesses in μm, based on age and sex

Age(years)	Overall		Male		Female	
	Mean	Standard deviation	Mean	Standard deviation	Mean	Standard deviation
3	531	30	528	25	536	38
4	536	32	538	29	534	34
5	539	32	538	33	539	32
6	543	32	547	32	538	31
7	549	28	558	27	542	26
8	551	28	554	26	549	29
9	547	31	546	33	548	30
10	553	29	553	31	554	28
11	547	37	545	29	549	45
12	561	31	562	31	559	32
13	564	30	566	30	561	29
14	558	33	559	32	556	35

Table S6. Distribution of anterior chamber depths in mm, based on age and sex

Age(years)	Overall		Male		Female	
	Mean	Standard deviation	Mean	Standard deviation	Mean	Standard deviation
3	2.72	0.23	2.77	0.24	2.63	0.18
4	2.72	0.24	2.80	0.23	2.64	0.23
5	2.81	0.23	2.86	0.21	2.76	0.24
6	2.89	0.23	2.94	0.23	2.84	0.22
7	2.98	0.25	3.06	0.22	2.92	0.26
8	3.02	0.26	3.06	0.28	2.98	0.23
9	3.09	0.22	3.09	0.22	3.08	0.22
10	3.10	0.23	3.15	0.22	3.05	0.23
11	3.16	0.22	3.22	0.23	3.10	0.20
12	3.25	0.26	3.30	0.24	3.17	0.26
13	3.20	0.26	3.21	0.26	3.17	0.26
14	3.25	0.25	3.28	0.24	3.17	0.26

Table S7. Distribution of lens thicknesses in mm, based on age and sex

Age (years)	Overall		Male		Female	
	Mean	Standard deviation	Mean	Standard deviation	Mean	Standard deviation
3	3.81	0.25	3.81	0.28	3.83	0.17
4	3.76	0.19	3.74	0.19	3.78	0.19
5	3.68	0.21	3.67	0.18	3.69	0.23
6	3.60	0.20	3.59	0.20	3.62	0.19
7	3.52	0.17	3.48	0.13	3.55	0.20
8	3.46	0.20	3.45	0.20	3.47	0.21
9	3.42	0.20	3.43	0.20	3.40	0.20
10	3.41	0.19	3.39	0.18	3.44	0.21
11	3.38	0.17	3.35	0.17	3.42	0.16
12	3.35	0.18	3.32	0.18	3.41	0.16
13	3.37	0.19	3.36	0.19	3.38	0.18
14	3.37	0.18	3.36	0.17	3.41	0.21

Table S8. Distribution of vitreous chamber depths in mm, based on age and sex

Age(years)	Overall		Male		Female	
	Mean	Standard deviation	Mean	Standard deviation	Mean	Standard deviation
3	15.05	0.63	15.23	0.67	14.74	0.45
4	15.12	0.72	15.39	0.69	14.86	0.66
5	15.39	0.62	15.55	0.60	15.23	0.61
6	15.69	0.72	15.92	0.72	15.41	0.62
7	16.01	0.71	16.23	0.80	15.83	0.57
8	16.35	0.85	16.51	0.93	16.19	0.73
9	16.61	0.93	16.73	0.91	16.49	0.94
10	16.76	1.16	17.10	1.19	16.45	1.05
11	17.11	1.09	17.34	1.03	16.85	1.11
12	17.41	1.08	17.67	0.99	16.95	1.09
13	17.45	1.14	17.67	1.11	17.06	1.09
14	17.38	1.11	17.66	1.07	16.71	0.89

Table S9. Distribution of axial length–corneal radius ratios, based on age and sex

Age(years)	Overall		Male		Female	
	Mean	Standard deviation	Mean	Standard deviation	Mean	Standard deviation
3	2.84	0.08	2.84	0.08	2.83	0.07
4	2.86	0.06	2.87	0.06	2.85	0.06
5	2.90	0.07	2.90	0.07	2.89	0.07
6	2.93	0.07	2.93	0.08	2.92	0.06
7	2.98	0.07	3.01	0.07	2.95	0.07
8	3.01	0.09	3.02	0.10	3.00	0.09
9	3.05	0.12	3.05	0.13	3.04	0.11
10	3.05	0.12	3.08	0.12	3.03	0.11
11	3.10	0.12	3.11	0.13	3.09	0.11
12	3.14	0.14	3.16	0.14	3.09	0.14
13	3.13	0.14	3.14	0.13	3.11	0.14
14	3.13	0.14	3.15	0.15	3.08	0.12

Table S10a. Lifestyle characteristics based on sex (preschool)

	Overall	Male	Female
Number	526	286	240
Age (years)	4.84 ± 0.88	4.86 ± 0.91	4.83 ± 0.85
Body mass index (kg/m^2)	15.79 ± 1.46	15.87 ± 1.35	15.70 ± 1.57
Time spent (min/day)			
Outdoors	73.7 ± 43.7	76.7 ± 44.1	70.1 ± 43.0
Watching television	92.3 ± 61.7	89.0 ± 59.8	96.3 ± 63.9
Use of digital devices	25.3 ± 37.9	28.0 ± 40.2	22.1 ± 34.8
Reading	30.8 ± 23.3	31.6 ± 25.0	29.7 ± 21.1
Number of myopic parents			
0 (none)	24.1%	24.5%	23.8%
1 (either)	35.2%	32.9%	37.9%
2 (both)	40.7%	42.6%	38.3%

The data are expressed as percentages or means ± standard deviations.

Table S10b. Lifestyle characteristics based on sex (elementary school)

	Overall	Male	Female
Number	543	269	274
Age (years)	8.40 ± 1.73	8.46 ± 1.73	8.33 ± 1.74
Body mass index (kg/m^2)	16.40 ± 2.10	16.62 ± 2.23	16.19 ± 1.94
Time spent (min/day)			
Outdoors	71.5 ± 45.7	78.0 ± 49.0	65.2 ± 41.2
Watching television	85.2 ± 58.1	87.0 ± 60.2	83.3 ± 56.0
Use of digital devices	40.5 ± 47.9	43.2 ± 51.9	37.8 ± 43.5
Reading	68.4 ± 65.6	66.4 ± 59.8	70.4 ± 70.8
Number of myopic parents			
0 (none)	15.5%	15.6%	15.3%
1 (either)	33.7%	30.9%	36.5%
2 (both)	50.8%	53.5%	48.2%

The data are expressed as percentages or means ± standard deviations.

Table S10c. Lifestyle characteristics based on sex (junior high school)

	Overall	Male	Female
Number	556	365	191
Age (years)	12.91 ± 0.81	12.93 ± 0.82	12.86 ± 0.80
Body mass index (kg/m^2)	18.73 ± 2.42	18.75 ± 2.56	18.69 ± 2.13
Time spent (min/day)			
Outdoors	72.2 ± 56.1	79.0 ± 57.2	59.2 ± 51.5
Watching television	80.7 ± 60.9	85.4 ± 63.9	71.8 ± 53.9
Use of digital devices	115.4 ± 88.5	122.5 ± 92.0	101.8 ± 79.9
Reading	74.7 ± 56.0	70.8 ± 54.2	82.1 ± 69.3
Number of myopic parents			
0 (none)	13.0%	12.3%	14.1%
1 (either)	45.1%	45.5%	44.5%
2 (both)	41.9%	42.2%	41.4%

The data are expressed as percentages or means ± standard deviations.

Table S10d. Lifestyle characteristics based on sex (overall)

	Overall	Male	Female
Number	1625	920	705
Age (years)	8.79 ± 3.52	9.12 ± 3.60	8.37 ± 3.37
Body mass index (kg/m^2)	17.00 ± 2.40	17.23 ± 2.49	16.70 ± 2.24
Time spent (min/day)			
Outdoors	72.4 ± 48.9	78.0 ± 51.0	65.2 ± 45.0
Watching television	86.0 ± 60.4	87.0 ± 61.5	84.6 ± 59.0
Use of digital devices	61.2 ± 74.0	70.0 ± 80.6	49.8 ± 62.6
Reading	58.4 ± 56.7	57.4 ± 52.0	59.7 ± 62.3
Number of myopic parents			
0 (none)	17.4%	17.1%	17.9%
1 (either)	38.1%	37.3%	39.1%
2 (both)	44.5%	45.6%	43.0%

The data are expressed as percentages or means ± standard deviations.
